# Perioperative Dystonia Mimicking Convulsion: Awareness Is the Key to Diagnosis

**DOI:** 10.7759/cureus.16676

**Published:** 2021-07-27

**Authors:** Bhanu P Swain, Deb Sanjay Nag

**Affiliations:** 1 Anesthesiology, Tata Main Hospital, Jamshedpur, IND

**Keywords:** dystonia, extrapyramidal side effects, metoclopramide, convulsion, airway obstruction, perioperative period

## Abstract

Dystonia, a variant of acute extrapyramidal symptoms (EPS), is a known side effect of neuroleptics. It is a rare but well-recognized complication in perioperative settings. Most of the reported cases have been linked to the perioperative use of metoclopramide. Dystonic reactions typically present as repetitive movements or abnormal posturing of the head, neck, and body. These reactions usually last only for a few minutes and are rarely lethal. However, occasionally they may present as sustained laryngopharyngeal muscle spasms, potentially leading to fatal airway obstruction. In this report, we present the case of an elderly female patient who developed a life-threatening episode of dystonia mimicking convulsion in the postoperative period. She had received metoclopramide as pre-anesthetic medication before surgery.

## Introduction

Acute-onset extrapyramidal symptoms (EPS) are drug-induced movement disorders characterized by involuntary muscle contractions of different body parts with varied presentations. The EPS spectrum includes three common manifestations: dystonia, akathisia, and parkinsonism [1].

Dystonia and akathisia are the most commonly reported extrapyramidal reactions in the perioperative period. Dystonia usually involves the head, eye (oculogyric crisis), neck (torticollis), trunk (opisthotonus), and, in rare cases, extremities. On the contrary, akathisia symptoms usually affect the extremities. Dystonic reactions are generally not life-threatening, but infrequently they may cause fatal upper airway obstruction due to severe laryngopharyngeal muscle spasms [2].

Dystonia or any EPS are classical adverse effects of neuroleptics [2]. However, several other medications like antidepressants, antiemetics, and antivirals have extrapyramidal side effects as well. Metoclopramide, ondansetron, and propofol are some of the offending medications associated with perioperative dystonia [3-7]. Patients with neurological disorders, those on antipsychotics, elderly patients, and children are at a higher risk of developing perioperative extrapyramidal reactions [8], and in these groups of patients, even a single dose of the offending drugs like metoclopramide may induce the symptoms.

There is often difficulty in differentiating acute dystonic reactions from convulsions because of their similar presentations [9]. Moreover, unfamiliarity with this rare complication could potentially result in a diagnostic dilemma. Proper clinical and drug history and paying close attention to the typical display of abnormal posturing or repetitive movements of body parts can provide the clues to reach a correct diagnosis.

We report a case of an elderly female patient who had an acute episode of dystonia with severe upper airway obstruction in the postoperative period after an uneventful orthopedic surgery under spinal anesthesia. The dystonic reaction was presumed to be induced by metoclopramide administered to the patient as a pre-anesthetic medication on the morning of surgery. Preoperatively, the patient was taking antidepressants and antivertigo medications.

## Case presentation

A 59-year-old female was admitted with a fractured neck of the right femur after a trivial fall at home. The patient had no history of any other associated injury. She was suffering from multiple comorbidities like hypertension, type II diabetes mellitus, bronchial asthma, major depression, and vertigo. She also had a history of stroke 15 years back. Preoperatively, she was on nifedipine 10 mg twice daily, acarbose 50 mg twice daily, aspirin 75 mg once daily, and atorvastatin 20 mg once daily. She was using inhalational bronchodilators on and off for asthmatic exacerbations. She was also being treated for depression and was on dosulepin 75 mg once daily and quetiapine 25 mg once daily. For vertigo, she was on betahistine 20 mg twice daily, along with flunarizine 10 mg once daily. On reviewing her medical chart, she was found to be suffering from restless leg syndrome, for which she was taking pramipexole 0.25 mg twice daily. There was no history of any drug allergies, and she denied any addiction or drug abuse. There was no previous anesthesia exposure.

She was scheduled for a modular bipolar hemiarthroplasty surgery. The preoperative blood investigations, chest X-ray, and electrocardiogram (ECG) were within normal limits, except for anemia with hemoglobin of 7.6 g/dl. The patient was classified as an American Society of Anesthesiologists (ASA) physical status classification III, and the surgery was planned under subarachnoid block. Preoperatively, she continued all her medications as per schedule till the morning of surgery, except the oral antidiabetic drugs. She received pantoprazole 40 mg and metoclopramide 10 mg orally two hours before surgery as per standard premedication protocol. Intraoperatively, she received a subarachnoid block with 3 ml of 0.5% hyperbaric bupivacaine. The anesthesia block produced a sensory deficit up to the T10 dermatome. There was a transient fall in blood pressure to a mean arterial pressure of 60 mmHg, which was managed with intravenous fluids and mephentermine injection (6 mg). Throughout the surgery, her vital signs remained stable. She was not administered any sedatives in the intraoperative period. She was fully conscious and oriented throughout the surgery.

After an uneventful intraoperative course, when she was being transported to the post-anesthesia care unit (PACU), she suddenly started extending her neck and back, clenched her teeth, and her eyeballs rolled upward. There was paradoxical respiration implying upper airway obstruction. She remained unresponsive to verbal commands as well. Her blood pressure increased to 180/90 mmHg, her heart rate was 112 beats per minute, and her oxygen saturation was 94% on room air. The airway obstruction was managed with difficulty by applying jaw thrust and insertion of the nasopharyngeal airway. She was provided supplemental oxygen by a Bain circuit. Midazolam 2 mg was immediately administered intravenously due to a suspicion of convulsion followed by phenytoin infusion. The patient’s symptoms and signs resolved after five minutes. She became fully conscious, but she was amnesic to the previous events. Her overall vitals were stable, and she was transferred to PACU. She again had a similar episode after about 15 minutes. However, this time it was mild and involved the eyes only, and lasted for one to two minutes without any signs of airway obstruction. The second episode resolved spontaneously without any further sequelae. Based on the typical symptomatology, a diagnosis of an extrapyramidal reaction (acute dystonia) triggered by metoclopramide was established. The phenytoin infusion was stopped as epilepsy was ruled out. A normal arterial blood gas ruled out any metabolic derangement. The patient did not have any further episodes of extrapyramidal reaction in the PACU, and she was shifted to a high dependence unit after four hours of observation. Postoperatively, a neurologist reviewed the case and corroborated our diagnosis based on the history and presentation. They augmented the dose of pramipexole she was taking earlier and advised her to follow up in the neurology outpatient department after discharge. The patient remained asymptomatic during the rest of her hospital stay.

## Discussion

EPS or drug-induced movement disorders are the most common side effects of dopamine receptor blocking agents. They are clinically manifested as sustained or repetitive muscle contractions of the face, head, trunk, and limbs. The first-generation antipsychotics (haloperidol and phenothiazines) are the most commonly implicated medications. Besides those, other antidopaminergic medications such as second-generation antipsychotics, antiemetics (metoclopramide, droperidol, ondansetron), tricyclic antidepressants (TCAs), selective serotonin reuptake inhibitors (SSRIs), certain antivirals, and antiarrhythmics are also reported to induce EPS [1]. The pathophysiology of EPS is not clearly understood. The most plausible hypothesis is an imbalance between dopamine and other neurotransmitters like acetylcholine, serotonin, and γ-aminobutyric acid (GABA) in the basal ganglia [10].

EPS is associated with a spectrum of manifestations. Dystonia, akathisia, and parkinsonism are acute manifestations of EPS. Tardive dyskinesia and tardive akathisia occur more chronically with long-term use of neuroleptics [1]. Dystonia is one of the most commonly reported acute-onset drug-induced EPS, and it is characterized by involuntary intermittent muscle contractions resulting in sustained abnormal posturing or repetitive movements affecting different body parts. The muscles of the neck (torticollis), jaw (trismus), eyes (oculogyric crisis), tongue (buccolingual crisis), and back (opisthotonus) are the most commonly affected areas. Extremities, abdominal wall, and pelvic muscles (tortipelvic crisis) are also infrequently involved [2]. Sometimes, dystonic symptoms may present with life-threatening severe upper airway obstruction due to the involvement of pharyngeal and laryngeal muscles [11]. Acute dystonia is often misdiagnosed as convulsion because of similar presentation, as observed in our case [12]. Akathisia is another drug-induced EPS characterized by both subjective and objective features affecting the lower extremity. Subjectively, the patient has a sense of restlessness and a compelling desire to move, which is manifested objectively as semi-purposeful repetitive movements like shuffling of legs, tapping of the foot, swinging, and shifting of weight from one foot to another. Akathisia is frequently misinterpreted as restless leg syndrome or anxiety disorder. Parkinsonism is more of a subacute extrapyramidal presentation. It is characterized by symmetrical rigidity of body parts, tremors, and slowing of motor function in patients [1].

The immediate management of acute dystonic reaction entails maintaining the airway in cases of airway obstruction and then providing medications to control the episode. Commonly used medications are antihistaminic agents (diphenhydramine, promethazine) and anticholinergic agents (benztropine, biperiden). Benzodiazepines (diazepam, midazolam) are also effective in aborting the acute reaction [13,14].

Undoubtedly, our patient was a high-risk candidate to develop EPS perioperatively. She was elderly, diabetic with a history of stroke, and she was also on medications with potential for extrapyramidal adverse effects (dosulepin, quetiapine, and flunarizine) [15-17]. Additionally, she was suffering from restless leg syndrome, a neurological movement disorder, before surgery. Pre-anesthetic medication with metoclopramide could have unmasked the underlying ailment leading to an acute dystonic reaction in the postoperative period.

Metoclopramide is an antiemetic commonly used in the perioperative period for prophylaxis as well as the treatment of postoperative nausea and vomiting. It is a cholinomimetic drug, and it exerts the clinical effect by peripheral and central antidopaminergic action. It induces extrapyramidal side effects by increasing the acetylcholine level in the central nervous system and antagonizing dopamine centrally, thereby causing an imbalance [8]. The side effects are not dose-related and often idiosyncratic. Even a single therapeutic dose can bring on EPS. The manifestation may occur within minutes or be delayed for 24-72 hours after metoclopramide administration [5]. The population at increased risk of development of metoclopramide-induced EPS are children, the elderly, patients with a history of neuropsychiatric disorder, patients with a history of EPS, patients on neuroleptics, and people with genetic polymorphism of CYP2DG gene [8]. The best possible way to prevent metoclopramide-induced EPS is to avoid the use of the drug in susceptible individuals. If metoclopramide is administered, especially intravenously, it must be given slowly over a duration of three minutes or more. The dose of the drug should be limited to less than 0.5 mg per day with a total duration of administration of not more than five days [18].

Apart from metoclopramide, drugs like ondansetron and propofol are also reported to cause dystonic reactions in the perioperative period [6,7]. Moreover, the complex interaction of multiple medications during anesthesia may occasionally lead to unpredictable central nervous system effects. Hence, an anesthesiologist needs to be highly suspicious of EPS to make a diagnosis. 

The current case was initially interpreted as convulsion because of the acute onset of symptoms. The diagnosis of acute dystonia was later confirmed based on the typical posturing of the head and neck, which is unusual in convulsion. Furthermore, the medical history (depression on antidepressants), presence of preoperative extrapyramidal movement disorder, and use of metoclopramide as premedication established the diagnosis. In this case, the acute dystonia was probably aborted by the administration of midazolam, though it was initially aimed at treating convulsion. The ideal first-line drug would have been an anticholinergic or an antihistaminic. However, midazolam is a readily available drug in most operation theatres, and it can be used to reverse acute extrapyramidal reactions. In the postoperative period, pramipexole was resumed (previously used by the patient for restless leg syndrome) after consulting a neurologist. A recent clinical trial has studied the efficacy of this drug in treating neuroleptic-induced EPS and found it to be effective [19]. However, caution should be exercised as the drug can reverse the effects of antidepressants and antipsychotics and aggravate the psychiatric manifestations.

## Conclusions

Acute extrapyramidal reactions like dystonia in the perioperative period can be overwhelming for an anesthesiologist. A lack of familiarity with the condition and its complex clinical manifestations may lead to the misdiagnosis of this uncommon scenario.

Neuropsychiatric illness, intake of neuroleptic medications, and prior movement disorders are some of the risk factors for the development of EPS. These factors need to be identified so that the perioperative caregiver would remain alert and avoid drugs aggravating EPS. As seen in this case, administration of metoclopramide to a susceptible patient may lead to a fatal dystonic reaction. Hence, this drug should be carefully prescribed, and preferably only to those patients with genuine indications, and it must be avoided in people at risk of developing EPS.

The medications preferred for treating drug-induced dystonia are diphenhydramine (antihistaminic), benztropine, or biperiden (anticholinergics). Benzodiazepines are also effective in aborting acute EPS episodes. The laryngopharyngeal muscles may be involved in severe cases, leading to life-threatening airway obstruction. Anesthesiologists should be aware of this fatal complication and address it immediately along with the administration of drugs.
